# Psychopathy, psychological distress, and treatment history among perpetrators of intimate partner femicide, homicide, and other violent crimes in Buenos Aires, Argentina

**DOI:** 10.1371/journal.pmen.0000064

**Published:** 2024-07-24

**Authors:** Martín Hernán Di Marco, Gergő Baranyi, Dabney P. Evans

**Affiliations:** 1 Department of Criminology and Sociology of Law, University of Oslo, Oslo, Norway; 2 University College London, London, United Kingdom; 3 Rollins School of Public Health, Emory University, Atlanta, Georgia, United States of America; University of North Carolina at Chapel Hill, UNITED STATES OF AMERICA

## Abstract

Intimate partner femicide—the killing of women based on their gender by their former or current partners—is a global long-standing manifestation of violence against women. Despite the enactment of femicide-specific laws in Latin America, femicide rates have remained relatively constant throughout the last decade. Often perpetrators are pathologized as suffering from mental illness, yet the data on their mental health status is still relatively unknown. Thus, more research is needed to understand the extent of poor mental health among these individuals. The purpose of this study was to compare levels of psychopathy, psychological distress, and treatment history among an all-male sample of intimate partner femicide perpetrators, male-male homicide perpetrators, and offenders convicted of other violent crimes in Buenos Aires, Argentina. This study utilized a cross-sectional survey based on data derived from a two-stage sampling strategy. The questionnaire included two standardized instruments for the measurement of psychopathy (revised Psychopathy checklist and the Levenson Self-Report Psychopathy scale) and one for general distress (Spanish version of 12-item General Health Questionnaire). The final sample included 205 prisoners including 68 intimate partner femicide perpetrators, 73 homicide perpetrators, and 64 individuals convicted of other violent crimes. There were no significant differences across these groups based on their socio-demographic characteristics. Participants did not differ in terms of their psychopathology; however, femicide perpetrators were statistically more likely to experience psychological distress. In addition, femicide perpetrators self-reported more prior episodes of mental and substance use treatments. The findings of increased psychological distress and prior mental health and substance use treatment among femicide perpetrators suggest that there may be missed opportunities for femicide prevention within the public health subspecialties of mental health and substance use disorders. This study suggests that femicide perpetrators likely require distinctive interventions, including self-assessments and harm mitigation tactics, to prevent their potential for femicide perpetration.

## Introduction

Femicide is a persistent public health and social issue with cultural, social, psychological, and institutional factors playing a role in its perpetration [1, 2]. Despite international efforts to confront such violence, the gender-based killing of women and girls remains a global challenge [3]. To date, two sectors have most frequently examined intimate partner femicide (IPF)—the most prevalent form of femicide [3]. Public health researchers have framed IPF as an extreme form of intimate partner violence comparing femicide perpetrators to perpetrators of non-lethal intimate partner violence. Meanwhile, criminologists examine IPF as a form of homicide comparing femicides to other forms of homicide or serious crime. Yet, as discussed by feminist scholars, femicide cannot be reduced to an individual-level action and instead requires systems-level intervention [4–6].

IPF is often portrayed as being perpetrated by individual bad actors whose acts are rare, likely because the crime is perceived as being socially egregious [7–10]. Yet closer examination of homicide data reveals persistent statistical trends: women are disproportionately killed by their intimate partners. One in every three female homicides is perpetrated by a current or former intimate partner accounting for roughly 48,000 deaths per year [2, 3]. In Latin America, reported femicide rates have remained relatively constant throughout the last decade, in spite of the enactment of femicide-specific laws in several countries of the region [3, 11]. In this context, the interest in understanding if perpetrators have specific features has gained recent attention [12–14]. Here we focus on IPF, the gender-based killing of women or girls by their past or current intimate partners.

Because there is significant overlap in the risk factors for lethal and non-lethal intimate partner violence perpetration, it remains difficult to distinguish the distinctive features of IPF—a specific subtype of femicide—and by extension the men who perpetrate it. Most work has focused on the development and validation of lethality risk assessments based on the observations of female partners or law enforcement actors in the context of non-lethal intimate partner violence. Similarly, other scholars have tried to differentiate between IPF perpetrators and perpetrators of non-lethal intimate partner violence [15]. When compared to men who used non-lethal violence against an intimate partner, Dobash, Dobash and Medina-Ariz found that IPF perpetrators were more likely to use a weapon, strangle, or sexually assault their partners [16]. While they were also more likely to have been violent in a prior relationship, they were less likely to have previously used violence against the woman they killed. They were also less likely to have been drunk at the time of the femicide [16]. Goussinsky found that lethal violence is usually a planned or premeditated event, motivated by despair and a desire to destroy a partner [15]. The feeling of being threatened and belittled was a common emotion reported by IPF perpetrators across several Latin American countries [17]. These studies suggest that the behaviors of IPF perpetrators may be distinguished in some ways from men who use non-lethal intimate partner violence. Yet the data on the mental health status of these men is mixed.

Based on a comparison of IPF perpetrators and men who do not exercise violence against women in Turkey, Tosun Altınöz et al. found that perpetrators were not significantly different in terms of individual characteristics (i.e., childhood traumas, psychopathology, and gender attitudes), however, migration history was linked to femicide [18]. As understood by the authors, migration not only implies a change of location, but a radical social change and a defense of male-chauvinistic views, as found by other studies [19, 20]. This study also underscores that a unique psychopathology for femicide perpetration may not exist.

Others, most often criminologists, have compared femicides to non-partner homicides or perpetrators of other crimes. Much of the femicide perpetration research has focused on demographic characteristics and criminality, with medical pathologization occurring frequently [21]. While the demographic, socioeconomic, and criminogenic heterogeneity of male batterers has been highlighted, the degree to which IPF perpetrators suffer from poor mental health still needs further scrutiny especially given that many of these homicides are followed by suicide, potentially confounding the relationship between IPF and perpetrator mental health [22–24].

Because some studies compare femicide perpetrators to perpetrators of non-lethal intimate partner violence, and others compare them to perpetrators of other crimes, the data on the mental health status of IPF perpetrators is inconclusive. Depression, anxiety, posttraumatic stress disorder, antisocial personality disorder, and borderline personality disorder have all been found to be correlated with intimate partner violence perpetration [25]. While prior research has attempted to identify specific mental health diagnoses in IPF perpetrators [26], there is still no clear picture of the extent of mental health problems in the total population of perpetrators. Within the limited research on IPF perpetrators, scholars have found two types of perpetrators: those with and without antisocial features [27–29]. One study in Spain used the Psychopathy Checklist-Revised (PCL-R) to evaluate the degree of psychopathy of femicide perpetrators, finding low rates among their sample, with higher scores correlating with shorter romantic relationships as compared to relationships without lethal violence [30]. Likewise, Echeburúa & Fernández-Montalvo found that psychopathic batterers did not perpetrate IPF more often than non-psychopathic batterers, instead the crime might be attributable to other variables [31]. By contrast, in a study about intimate partner violence perpetration in Portugal, Cunha et al. found that PCL-R scores positively predicted the frequency of violence, more than other criminal variables [32]. To this end, some intimate partner violence recidivism assessment tools have included the PCL-R as part of their assessment of likely although these tools were not designed to assess femicide risk, again complicating the ability to differentiate between men likely to perpetrate lethal versus non-lethal violence.

Overall, few studies have focused on IPF perpetrator’s psychological or mental distress, and the results have varied. Belfrage and Rying compared the characteristics of IPF perpetrators to other homicide perpetrators in Sweden concluding that psychopathy is not overrepresented among IPF perpetrators; instead, depressive disorders were most common [33]. In comparing mental disorders among femicide perpetrators and male-male homicide perpetrators, Caman et al. found that, regardless of the type of homicide, one-third of the participants were diagnosed with mental disorders during their life span, yet low rates of serious or severe mental illness were found in both groups when aggregating lifetime diagnoses and diagnoses during the commission of the crime [23].

While the public and lay publications have taken for granted the fact that perpetrators have mental health problems, the evidence is far from settled. Femicide perpetration in general and particularly the mental health status and internal mental processes of femicide perpetrators remain under-researched [12]. We hypothesize that IPF perpetrators will not report experiencing higher rates of mental health problems, psychological distress, and psychopathy relative to other criminals. The hypothesis originated from our extensive qualitative fieldwork experience [17, 34, 35] and close readings of literature on femicide and homicide perpetration, including insights into the mental health, psychological distress, and psychopathy of such perpetrators. The purpose of this study was to compare levels of psychopathy, psychological distress, and treatment history among a sample of IPF perpetrators, male-male homicide perpetrators, and other offenders convicted of violent crimes in Buenos Aires, Argentina.

## Methods

### Participants and design

This study utilized a cross-sectional design. Participants included incarcerated cisgender men at four correctional facilities in Metropolitan Buenos Aires, Argentina. These facilities represent approximately the 27% of the total inmate population of the province. Participants were convicted for the perpetration of IPF, male-male homicide, or other crimes (robbery and/or assault excluding rape or sexual assault). The latter group was chosen to encompass men who were not charged with gender-based violence of any sort. This decision was made based on evidence indicating differences in socio-demographic and criminogenic characteristics between intimate partner violence perpetrators and individuals convicted of other types of crimes [36]. All these crimes were defined according to the legal definitions established in the Argentinean National Prosecution Statistical System (known in Spanish as SNEEP). The primary distinctions between homicide and IPF are the gender of the victim, and perpetration of the crime by a past or current intimate partner. Male-male homicide perpetrators were included in the sample as a criminal group comparable to IPF perpetrators as both crimes involve murder. The group of other violent crime perpetrators served as the control group. This approach is supported by criminological studies indicating differences in the criminal social identity between murderers and other types of offenders [23, 37].

### Instruments and outcomes

We developed a quantitative questionnaire using items adapted from existing instruments and novel items for administration among perpetrators of lethal and non-lethal crimes to explore three domains of inquiry—psychopathy, psychological distress, and treatment history.

The questionnaire covered topics that are often explored relative to IPF including sociodemographic measures, violence exposure, past violence perpetration within the index relationship, and past crime. A pilot stage was carried out, with a sample of 15 individuals (five from each perpetrator group), to assess the participant burden and participants’ response to and understanding of the questions.

### Psychopathy

Two existing instruments were used to assess psychopathy, the revised Psychopathy Checklist and the Levenson Self-Report Psychopathy scale.

The revised Psychopathy Checklist (PCL-R) is a 20-item scale designed to assess psychopathy in a forensic setting [38, 39]. This psychopathology scale is viewed as the gold standard for predicting violent behavior. Each item is scored on a 3-point scale (0 = does not apply, 1 = somewhat applies, 2 = definitely applies) indicating the degree of alignment between item description and personality or behavior. It is a valid and reliable scale widely used among incarcerated populations [39, 40], including previous use in Argentina [41, 42]. Potential summary scores range from 0 and 40. We calculated scores for the domains of emotional detachment (e.g., grandiose sense of self-worth, pathological lying, lack of remorse or guilt) and antisocial behavior (e.g., impulsivity, irresponsibility, juvenile delinquency, with 8 and 9 items belonging to each domain, respectively [39]. Cronbach’s Alpha was acceptable for the total scale (α = 0.74), and good/excellent for emotional detachment (α = 0.87) and antisocial behavior (α = 0.94) subscales.

The Levenson Self-Report Psychopathy Scale (LSRP) scale was developed to assess primary and secondary psychopathic features among general, non-institutionalized populations [43]. The instrument consists of 26 items, with responses assessed on a 4-point Likert scale. For our purposes, we modified the original Likert scale where responses ranged from strongly agree to strongly disagree to a scale where responses ranged from agree to disagree and responses 2 and 3 on the scale aligned with slightly agree and slightly disagree respectively. We made this alteration to simplify the scale to account for administration to our sample who may lack familiarity with survey design and who may also have a lower literacy level. Summary scores may range between 0 and 78. The scale’s validity and reliability have previously been assessed [44]; similar to PCL-R, the LSRP consists of two domains; 16 items capture primary psychopathy with manipulative, selfish, and irresponsible behaviors and attitudes, and 10 items capturing secondary psychopathy with impulsive and antisocial lifestyle [43]. Internal consistency was good for the total (α = 0.86) scale and excellent for primary (α = 0.96) and secondary (α = 0.96) psychopathy. For three participants responses of neutral were utilized which was not in keeping with our scale modifications, therefore we excluded these participants from the analytical sample.

### Psychological distress

The Spanish version of the 12-item General Health Questionnaire (GHQ-12) was administered by interviewers to assess psychological distress [45, 46]. The scale has shown adequate validity and reliability among adult populations in Argentina, Colombia and Spain [47–50]. It has also been used among incarcerated populations worldwide [51, 52]. GHQ-12 has 12 items, six positively and six negatively coded, each with four Likert-style response options [53]. Summary scores range between 0 and 36 with higher values indicating higher psychological distress.

### Treatment history

Treatment history for mental health and substance use problems were explored via two original yes/no binary questions: *“Have you ever been treated (medication*, *hospitalization*, *therapy*, *etc*.*) for mental health problems*?*”* and *“Have you ever been treated (medication*, *hospitalization*, *therapy*, *etc*.*) for alcohol or drug use problems*?*”* Responses were coded as either having treatment history (0) or not (1).

### Procedure

The survey sample was a two-stage design in which the prisons were selected in the first stage, and prisoners within the sampled facilities were selected in the second stage. First, four maximum-security prison facilities in Metropolitan Buenos Aires were purposively selected, based on permissions granted by penal authorities. These facilities include those with the highest number of inmates in the region. Second, participants were recruited using random sampling until COVID-19 restrictions were implemented terminating the fieldwork. Prison authorities provided a list of all the cisgender men incarcerated for the previously established crimes and authorized contact with them during their scheduled activities (such as work, classes, or sports) or assisted in organizing meetings with them (for instance, in the visitor’s area). Participants were randomly selected from the list of each group (IPF, homicide, and other violent crimes) by drawing one case per group per round. No specific inclusion/exclusion criteria were set beforehand, apart from their gender, conviction status, and willingness to participate. In the case of femicide, only cases where the convicted man had been charged with the homicide of a partner or ex-partner were included. This sampling strategy purposively oversampled IPF perpetrators to facilitate comparison between the groups.

Within each sample strata or population group, the enrollment rates were 87% (homicide), 94% (IPF), and 79% (other violent crimes). Refusals to participate were noted in field notes. These refusals were often due to a lack of interest in academic research, a reluctance to discuss their private lives, and a general distrust of external researchers. These reasons mirror the motivations identified in previous studies on recruitment in Argentina [54].

Prior to data collection, participants were informed of the purpose of the study and gave their informed consent. Given that our sample population is incarcerated we took extra precautions to ensure that participation was voluntary including a risk-benefit analysis and collaborative responsibility approach [55, 56]. Interviews took place in private spaces within the facilities, such as classrooms, visitor rooms, multipurpose rooms, and internal patios. As a general principle, interviews were not conducted in the presence of other inmates or prison staff to avoid exposing participants to potential internal conflicts.

Surveys were administered in person between June 17^th^ and November 30^th^, 2020; five trained male enumerators read each survey item to participants and recorded their responses on a paper survey. Given the highly stigmatized nature of the crimes for which the interviewed men were convicted, we believed that conducting interviews with men would establish a stronger rapport. For the revised Psychopathy Checklist (PCL-R), two trained psychologists who were members of the research team, collaborated with the forensic psychologists of each facility, using interview and case file information to complete the checklist.

Previously arranged interviews which were disrupted by COVID mitigation efforts were conducted by phone (n = 114). Interviews lasted between 35–60 minutes. Phone conversations were scheduled in advance to enable participants to use the phones available in the educational facilities of the prisons. This arrangement ensured that they could have private conversations in a room without other people present. Data were subsequently entered into an Excel spreadsheet and later exported into R, version 4.2.1 for analysis [57].

### Statistical analysis

The study explored whether participants convicted of femicide differed in sociodemographic characteristics, psychopathy, psychological distress, and treatment history from individuals convicted of homicide, or other types of violent crime. We assessed significant differences in key covariates between the three groups of conviction using one-way ANOVA for continuous and chi-square tests for categorical (including binary) variables.

We ran multivariate regression analyses to investigate whether potential differences in the mental health status between participants convicted for different types of crime were explained by potential confounders and study design variables. Linear regressions were performed for GHQ-12, PCL-R and LSRP (effect estimates were expressed as unstandardized coefficients [b]); multivariate Poisson regression with log link was run to assess differences by the presence or absence of mental health and substance use treatment history.

Here we present models by two steps of covariate adjustment (Table 1). In Model 1, we adjusted for age in years (at the time of interview) and study design variables. Despite interviewers receiving the same training and use of standardized instruments we considered the possibility that there were unmeasured differences in the ways interviewers conducted interviews. Similarly, we assumed differences between the four correctional institutions and mode of interview (face-to-face versus phone).

**Table 1:** Model 1 and 2 developed for the study.

**A)**	**Model 1** ^**a**^			**Model 2** ^**b**^		
	b	95% CI	p-value	b	95% CI	p-value
1. Psychological distress (GHQ-12)						
Homicide	-2.511	-4.832, -0.189	0.034	-2.506	-4.617, -0.395	0.020
Femicide	1.624	-0.754, 4.002	0.180	0.967	-1.217, 3.152	0.383
Other violent crimes	ref			ref		
2. Psychopathy (PCL-R)						
Homicide	0.842	-1.393, 3.078	0.458	0.600	-1.645, 2.844	0.599
Femicide	0.969	-1.321, 3.260	0.405	0.723	-1.600, 3.046	0.540
Other violent crimes	ref			ref		
Emotional detachment						
Homicide	0.988	-0.483, 2.458	0.187	0.892	-0.598, 2.382	0.239
Femicide	0.037	-1.470, 1.544	0.961	-0.155	-1.697, 1.387	0.843
Other violent crimes	ref			ref		
Antisocial behavior						
Homicide	-0.269	-2.240, 1.702	0.788	-0.386	-2.393, 1.620	0.704
Femicide	0.694	-1.325, 2.713	0.499	0.653	-1.423, 2.729	0.536
Other violent crimes	ref			ref		
3. Psychopathy (LSRP)						
Homicide	0.513	-4.555, 5.580	0.842	0.071	-4.961, 5.103	0.978
Femicide	0.758	-4.435, 5.950	0.774	0.264	-4.943, 5.471	0.921
Other violent crimes	ref			ref		
Primary						
Homicide	-0.162	-5.650, 5.326	0.954	-0.353	-5.865, 5.159	0.900
Femicide	0.907	-4.716, 6.529	0.751	0.941	-4.762, 6.644	0.745
Other violent crimes	ref			ref		
Secondary						
Homicide	0.675	-2.969, 4.319	0.715	0.424	-3.234, 4.082	0.819
Femicide	-0.149	-3.882, 3.584	0.937	-0.678	-4.462, 3.107	0.724
Other violent crimes	ref			ref		
**B)**	**Model 1** ^**a**^			**Model 2** ^**b**^		
	RR	95% CI	p-value	RR	95% CI	p-value
4. Mental health treatment history						
Homicide	0.33	0.11, 0.88	0.036	0.33	0.11, 0.89	0.037
Femicide	3.76	2.09, 7.28	<0.001	3.87	2.12, 7.56	<0.001
Other violent crimes	ref			ref		
5. Substance use treatment history						
Homicide	1.01	0.50, 2.10	0.972	1.06	0.52, 2.20	0.875
Femicide	3.25	1.82, 6.16	<0.001	3.52	1.96, 6.72	<0.001
Other violent crimes	ref			ref		

Models were fitted with A) multivariate linear regression (expressed in unstandardized coefficients [b]) and B) multivariate Poisson regression with log link (expressed in Risk Ratios [RR]).

^a^ Model 1: controlled for age, type of interview, prison facility and interviewer.

^b^ Model 2: Model 1 + country of origin, highest education, make ends need (before the crime), and history of conviction.

In Model 2, we further adjusted for key covariates likely biasing the association between type of crime committed and mental health status. These included: country of origin (Argentina, other countries), highest education (primary, secondary, tertiary), “making ends meet” before being imprisoned (easily, fairly easily, with some difficulty, with great difficulty), and prior convictions (yes, no). High correlation between covariates might introduce bias in estimating the association of interest; therefore, we calculated variance inflation factors which suggested no multicollinearity (VIF<2.01).

### Ethical considerations

This project was approved by the Ethical Review Board “Vicente Federico de Giúdice” of Posadas Hospital, an Argentinian authority on research ethics. The study falls under a larger body of research, the Narratives of life and death Project (PRI DAR 2938/20). Emory University’s Institutional Review Board also granted an exemption for the work developing the questionnaire. Written, informed consent was obtained in Spanish from all participants before the questionnaire was administered. Following the established research guidelines, participants were given informed consent documents that described the purpose of the study, length of participation, and informed them that participation was entirely voluntary. Participants had the option to withdraw from the study at any time, and non-participants received the same treatment as participants. To minimize potential conflicts or feelings of coercion, all communications were conducted individually, and group meetings were avoided.

## Results

The final sample included 205 prisoners including 68 IPF perpetrators, 73 homicide perpetrators, and 64 individuals convicted for other violent crimes. There were no significant differences across femicide, homicide, and perpetrators of other crimes based on their socio-demographic characteristics including age, educational attainment, ability to make ends meet before the crime, and prior criminal history. However, femicide perpetrators were less likely to have a prior criminal conviction relative to perpetrators of homicide and other crimes. Perpetrators of all types were in their mid-late 20s with over 90% being born in Argentina (Table 2).

**Table 2:** Sociodemographic characteristics of Argentinean perpetrators of femicide, homicide and other violent crimes.

Characteristics	Femicide (*n* = 68)	Homicide (*n* = 73)	Other violent crimes (*n* = 64)	p-value
Age, mean (SD)	27.4 (11.2)	29.4 (10.9)	30.7 (11.8)	ns
Country of origin, n (%)				ns
Argentina	65 (95.68)	70 (95.98)	60 (93.8%)	
Others (Bolivia, Paraguay)	3 (4.4%)	3 (4.1%)	4 (6.2%)	
Highest level of education, n (%)				ns
Primary	30 (44.1%)	37 (50.7%)	35 (54.7%)	
Secondary	26 (38.2%)	23 (31.5%)	20 (31.2%)	
Tertiary	12 (17.6%)	13 (17.8%)	9 (14.1%)	
Make ends meet (before the crime), n (%)				ns
Easily; fairly easily	13 (19.1%)	9 (12.3%)	7 (10.9%)	
With some difficulty	10 (14.7%)	10 (13.7%)	11 (17.2%)	
With great difficulty	45 (66.2%)	54 (74.0%)	46 (71.9%)	
History of prior conviction, n (%)	20 (29.4%)	29 (39.7%)	32 (50.0%)	ns

The mean values of the PCL-R and LSRP were 17.6 (range = 3 to 30; SD = 6.5) and 34.1 (range = 3 to 67; SD = 14.9) respectively. The mean of GHQ-was 19.9 (range = 6 to31; 7.0 SD), and 180 (87.8%) respondents had a score above 11 for GHQ-12 likely indicating the presence of emotional disorders.

Participants did not differ in terms of their psychopathology across groups; however, femicide perpetrators had higher GHQ-12 scores meaning they were statistically more likely to experience psychological distress (Table 3). Across all participants, mental health treatment history was reported by 70 (34.1%), substance use treatment history by 80 (39.0%) participants. In addition, while femicide perpetrators self-reported previous episodes of mental (76.5%) and substance use (72.1%) treatments, these were much lower among homicide perpetrators (6.8%) or individuals convicted for other violent crimes (20.3%).

**Table 3:** Mental characteristics (psychopathy, psychological distress, and treatment history) of Argentinean perpetrators of femicide, homicide and other violent crimes.

Characteristics	Femicide (*n* = 68)	Homicide (*n* = 73)	Other violent crimes (*n* = 64)	p-value
PCL-R, mean (SD)	18.0 (6.53)	17.7 (6.72)	17.2 (6.41)	ns
Emotional detachment, mean (SD)	6.34 (4.20)	7.14 (4.51)	6.30 (4.08)	ns
Antisocial behavior, mean (SD)	8.93 (5.97)	7.99 (5.62)	8.33 (5.62)	ns
LSRP, mean (SD)	34.2 (14.6)	33.8 (15.6)	34.2 (14.5)	ns
Primary psychopathy	22.6 (16.3)	21.5 (15.8)	22.2 (15.4)	ns
Secondary psychopathy	11.6 (10.8)	12.3 (10.6)	12.0 (10.4)	ns
GHQ-12 score, mean (SD)	21.9 (7.05)	17.8 (6.14)	20.2 (7.46)	<0.01
History of mental health treatment, n (%)	52 (76.5%)	5 (6.8%)	13 (20.3%)	<0.001
History of substance abuse treatment, n (%)	49 (72.1%)	17 (23.3%)	14 (21.9%)	<0.001

Significant differences between groups were tested using one-way ANOVA for continuous and chi-square tests for categorical variables.

Abbreviations: GHQ-12 = 12-item General Health Questionnaire; LSRP = Levenson Self-Report Psychopathy Scale; ns = not significant; SD = standard deviation; PCL-R = Psychopathy Checklist-Revised.

In multivariate models, we explored whether type of crime was associated with psychopathology, psychological distress, and self-reported treatment history after accounting for relevant covariates.

### Psychopathology

After adjusting for Model 1 covariates (i.e., age, study design variables), we did not observe any differences in terms of psychopathy between the different types of prisoners, which remained the same in Model 2 after further adjusting for sociodemographic covariates (i.e., country of origin, highest education, making ends meet before to the crime, prior convictions). For femicide perpetrators, the score values for the total PCL-R scale (b = 0.723, 95% CI—1.600, 3.046; p = 0.540), as well as for the emotional detachment (b = -1.155, 95% CI -1.697, 1.387; p = 0.843) and antisocial behavior subscales (b = 0.653, 95% CI -1.423, 2.729; p = 0.536) did not differ from those among homicide perpetrators. Similarly, we did find any differences in the total LSRP (b = 0.264, 95% CI -4.943, 5.471; p = 0.921), or in the primary (b = 0.941, 95% CI -4.762, 6.644; p = 0.745) or secondary psychopathy subscales (b = -0.678, 95% CI -4.462, 3.107; p = 0.724) based on femicide and homicide perpetration.

### Psychological distress

Femicide perpetrators had higher scores of GHQ-12 in the Model 1. In the fully adjusted model (Model 2) in comparison to other violent crimes, males convicted for femicide had higher scores, 0.967 on the GHQ-12 scale (95% CI: -1.217, 3.152; p = 0.383); individuals convicted for homicide reported more psychological distress relative to individuals convicted of other crimes (b = -2.506, 95% CI: -4.617, -0.395; p = 0.020) (Table 1).

### Treatment history

Finally, being imprisoned for femicide was associated which much higher risk of self-reporting mental health and substance use treatment. In the fully adjusted model, femicide perpetrators were more than three times as like as individuals convicted for violent crimes to self-report mental health treatment (3.87; 95% CI: 2.12, 7.56; p = <0.001); Femicide perpetrators also had increased relative risk of reporting substance use treatment history 3.52 (95% CI: 1.96, 6.72; p<0.001) (Table 1).

## Discussion

Our objective was to contribute to the academic literature regarding the individual characteristics of femicide perpetrators, by comparing their levels of psychopathy, psychological distress, and treatment history relative to perpetrators of homicide and other serious crimes in Buenos Aires, Argentina. These data may contribute towards disentangling questions about the risk factors—including mental health status—for lethal violence and upstream prevention strategies.

First, among our sample of demographically similar Latin American perpetrators of femicide, homicide, and other violent crimes we did not find significant differences in terms of their psychopathology, antisocial behavior or emotional detachment. The average values for psychopathology (PCL-R) were similar to Santos-Hermoso [30]. The lack of significant differences between the compared groups coincides with several studies in other regions [18, 30, 31, 33]. Dobash, Dobash and Medina-Ariza found IPF perpetrators do not report significantly different socio-demographic or mental health characteristics when compared to perpetrators of non-lethal intimate partner violence [16]; this comparison group differs from our comparison with homicide and perpetrators of other crime, underscoring the challenges in comparing IPF perpetrators to others (either intimate partner violence or other homicide and other crime perpetrators). As the first study to analyze these variables in Latin America, we interpret these findings as further supporting the notion that there are no psychopathology diagnoses for perpetrators of IPF. Thinking of femicide perpetrators as men with a singular distinguishable mental health diagnosis or pathology likely undermines our efforts at identifying them, and thus our ability to prevent femicide.

Second, we found higher psychological distress (GHQ-12 scores) among femicide perpetrators, although the reason for this distress is unclear. The fact that this group is statistically more likely to experience psychological distress was similarly found in other studies [33]. The elevated scores in this aspect may be explained by the increased perceived contextual and interpersonal pressures experienced by perpetrators, such as economic strain, fear of losing their partner, and general social anxiety [17, 23, 34]. Qualitative analysis of IPF perpetrator’s rationality aligns with this interpretation [12]. We also found considerably higher self-reported prior mental health and substance use treatment among femicide perpetrators. The fact that femicide perpetrators are more than three times as like as individuals convicted for other crimes to self-report mental health treatment is in line with many studies on risk factors of intimate partner violence and femicide specifically [3, 58, 59]. The findings of increased psychological distress and prior mental health and substance use treatment among femicide perpetrators suggest that there may be missed opportunities for femicide prevention in the fields of mental health and substance use disorders.

Based on a meta-analysis, Spencer et al. argue that timely and comprehensive violence screening is crucial for preventing various forms of intimate partner violence [60]. Professionals in mental health and substance use disorder fields can play a pivotal role in identifying and intervening with men seeking support. However, men are generally less inclined to seek mental health care, indicating the necessity for proactive efforts beyond these sectors. Given that IPF perpetrators reported higher treatment levels, it’s crucial to identify potential intervention points within social systems such as healthcare facilities, psychologists, and counselors. Screening, risk assessment, and intervention strategies should involve a broad range of professionals equipped to address the immediate mental, social, and legal aspects of this situation to detect potential femicide perpetration risks. Tools that differentiate between men likely to commit lethal versus non-lethal violence, along with corresponding interventions, are essential in this context.

Thirdly, femicide perpetrators reported fewer prior convictions. While not statistically significant—or included as a confounder for the other variables—this aspect is fundamental for the purposes of identifying potential perpetrators, as it would imply the possibility of early intervention, for instance regarding gender norms [61]. Since both male-male homicide and the other violent crimes clustered in this study are more associated with criminal careers (i.e., property crimes, drug dealing, etc.) and previous contact with penal institutions [29], it seems likely that that men who commit IPF do not have the same institutional pathways or systems-level engagement, perhaps even if they are also perpetrators of intimate partner violence. This finding underscores difficulties in detecting men at risk of femicide. IPF perpetrators may possess social skills, such as manipulating their partners to avoid legal action, and have networks of peers who support their coercive control before the femicide [29, 62]. These factors contribute to their ability to avoid engagement with law enforcement. As a result, upstream approaches for prevention are necessary since these men may evade or otherwise not be engaged with the criminal justice system. Femicide perpetrators likely require distinctive interventions—including self-assessments and harm mitigation tactics to prevent their potential for femicide perpetration.

## Limitations

The present study is not without limitations. First and foremost, the challenges when conducting first-hand quantitative research in prison settings inherently imply institutional barriers, necessary agreement with penal institutions and sometimes being subjected to informal regulations within each facility (access blocks to certain areas, social dynamics influencing participant’s behavior). While we aimed to implement a recruitment strategy that prioritized the safety, trust, and comfort of potential participants, we acknowledge that we are unaware of factors influencing their decision to participate, such as concerns about potential retaliation from prison staff (e.g., informal punishments for "speaking out of turn"). However, the high enrollment rates (87%, 94%, and 79%) may suggest otherwise. Moreover, institutional permissions have an impact in the feasibly of using randomized samples, calculating the power of statistical tests, as mentioned in other studies [23, 30, 63]. In our prior systematic review of femicide perpetrators, we found few studies that used randomized samples [12].

Second, the sampling strategy limits the potential generalization of the results. The fact that the first stage of the sampling included a purposeful selection of institutions has potential implications for the data collected. However, since the institutions where fieldwork was conducted house a large number of imprisoned people, we believe this limitation was partially overcome. Likewise given that femicide is a relatively rare event the inclusion of comparably sized groups of perpetrators of femicide, homicide, and other crimes is not unimportant.

Third, the fact that most of the variables are self-reported (with the exception of the PCL-R) might imply certain biases, such as that answers may be exaggerated, or participants may be embarrassed or unwilling to talk about certain private topics. However, the high response rates could be interpreted as an interest in participating, which could be explained by dominant ideas in people in prison about talking about their lives [54].

Fourth, the lack of prior studies focusing on the mental health of imprisoned population and research using the scales applied here to a largely Argentinean population make it challenging to establish comparisons. Our results, however, show lower rates of psychological distress in comparison to average scores in other studies focused on imprisoned populations [51, 64]. Unfortunately, due to the lack of studies applying GHQ-12 on general Argentinean population, no further comparison can be made. Furthermore, the dearth of these data for the general population limits a possible understanding of the national frame for mental health measurements.

Fifth, due to the cross-sectional design of this project, this study cannot establish causal conclusions. Specifically, this means we cannot state whether the mental health problems we examined were already present before committing the crime, emerged after the event, or even developed during imprisonment.

Sixth, it is worth mentioning that a proportion of the femicide cases are unsolved or wrongly classified in Argentina, as in other countries [3, 65]. Consequently, some femicide perpetrators could have either been wrongly classified (i.e. as homicide perpetrators) or not imprisoned at all meaning that a subsample of femicide perpetrators may not be represented.

Lastly, the high rates of IPF-suicide (calculated to be 30% of the cases overall) could be considered a limitation [3]. Particularly, because suicide is linked to mental health status, this could potentially confound the relationship between IPF and the variables measured [23, 24].

Despite these limitations, studies such as the one presented here are useful as they acknowledge that there is not a completely ideal methodological scenario to analyze femicide perpetrators in the current institutional and legal scenario, in which this crime has been incorporated into the penal frameworks in relatively recent years. Furthermore, in spite of the obstacles, the present study identifies data on how common, or rare, various mental disorders are in a comparative sample of perpetrators of femicide, homicide, and other crimes providing windows of opportunity for prevention.

## Conclusion

Deciphering the extent to which mental health characteristics have a role in the perpetration of femicide is crucial to understand not only the risk factors, root causes, and triggers of this crime, but also to plan adequate prevention strategies. Our study indicated that there are no significant differences in terms of sociodemographic characteristics or psychopathology when comparing perpetrators of IPF, homicide, and other serious violent crimes. Femicide perpetrators report higher levels of prior mental health and substance use treatment, and psychological distress—although the temporal nature of this distress is unclear. These findings suggest potential missed opportunities for screening, risk assessment, and interventions prior to the crime within the fields of mental health and substance use disorders. The study suggests that femicide perpetrators likely need tailored interventions, including self-assessments and harm mitigation tactics, to prevent their potential for femicide perpetration.
